# Interactions between nuclear and mitochondrial SNPs and Parkinson’s disease risk

**DOI:** 10.1016/j.mito.2022.02.002

**Published:** 2022-02-12

**Authors:** Sarah J. Pickett, Dasha Deen, Angela Pyle, Mauro Santibanez-Koref, Gavin Hudson

**Affiliations:** a Clinical and Translational Sciences Institute, Faculty of Medical Sciences, Newcastle University, Newcastle Upon Tyne NE2 4HH, UK; b Biosciences Institute, Faculty of Medical Sciences, Newcastle University, Newcastle Upon Tyne NE2 4HH, UK

**Keywords:** Case only design, Parkinson’s disease, Mitonuclear combinations

## Abstract

Interactions between the products of the nuclear and mitochondrial genomes are critical for the function of most eukaryotic cells. Recently the introduction of mitochondrial replacement therapy has raised the question of incompatibilities between mitochondrial and nuclear variants, and their potential influence on the genetic makeup of human populations. Such interactions could also contribute to the variability of the penetrance of pathogenic DNA variants. This led us to investigate the frequencies of combinations of nuclear and mitochondrial SNP alleles (mitonuclear combinations) in healthy individuals (n = 5375) and in a cohort of patients with Parkinson’s disease (PD, n = 2210). In the unaffected population, we were not able to find associations between nuclear and mitochondrial variants with a false discovery rate below 0.05 after accounting for multiple testing (i. e., the number of combinations examined). However, in the PD cohort, five combinations surpassed this threshold. Next, after combining both cohorts, we investigated whether these associations were modulated by disease status. All five combinations were significant (p < 10 ^−3^ for all tests). These combinations also showed significant evidence for an effect of the interaction between the mitochondrial and nuclear variants on disease risk. Their nuclear components mapped to *TBCA, NIBAN3*, and *GLT25D1* and an uncharacterised intergenic region. In summary, starting from a single cohort design we identified combinations of nuclear and mitochondrial variants affecting PD disease risk.

## 1 Introduction

The physiological function of eukaryotic cells requires contributions from both mitochondrial and nuclear gene products (Chinnery and Hudson, 2013). Sequence variation in the nuclear (nDNA) or mitochondrial (mtDNA) genome can lead to phenotypic changes (Chinnery and Hudson, 2013). Diseases linked to specific mtDNA variants, such as mt.3243 or mt.11778 (Chinnery and Hudson, 2013) show wide differences in penetrance and clinical presentation (Lopez Sanchez et al.,2021; Pickett, 2018), with studies indicating that nDNA contributes to these differences (Pickett, 2018; Hudson et al., 2005).

Common mtDNA variants have been repeatedly associated with complex disease, particularly diseases involving neurological degeneration (Hudson et al., 2013; Hudson et al., 2014; Ghezzi et al., 2005; Tranah et al., 2015; Vyshkina et al., 2005; Rollins et al., 2009; Gonçalves et al., 2018). However, beyond a few examples such as Parkinson’s disease (PD) (Marom et al., 2017; Hudson et al., 2013; Hudson et al., 2014; Ghezzi et al., 2005), such associations have proved difficult to replicate (Samuels et al., 2006). This could reflect the small effect sizes associated with mtDNA variants (Hudson et al., 2013; Hudson et al., 2014) or modulation of their effects by nDNA variation. Recently the introduction of mitochondrial replacement therapy (Kang et al., 2016; Yamada et al., 2016; Hyslop et al., 2016), has raised the question of whether mitonuclear incompatibilities exist (Yamada et al., 2016; Wei, 2019), and more generally, the extent to which these incompatibilities may influence health (Kivisild, 2015).

Within a population, the frequencies of combinations of nuclear and mitochondrial variants will reflect population structure as well as selection. In a cohort of patients affected by a specific disease, and where disease risk is influenced by the interaction from mitochondrial and nuclear variants, depletion or enrichment of corresponding combinations will be detectable as deviations from the frequency expected given the frequencies of the individual variants in the cohort. This has motivated using measures of association between alleles to detect such interactions (as ‘mitonuclear associations’). However, these associations could also reflect admixture or other forms of population stratification. This is of particular interest because mitochondrial variants have been extensively used to track the migration of populations and ancestry (Sloan et al., 2015).

Mitonuclear associations have been repeatedly investigated (Yamamoto et al., 2020; Yonova-Doing et al., 2021; van Oven and Kayser, 2009). An analysis of Human Genome Diversity Project data identified significant, albeit weak, mitonuclear associations through linkage disequilibrium (Yamamoto et al., 2020). However, recent work in the Japanese population found no evidence for such associations (Yonova-Doing et al., 2021). The importance of considering population structure was also highlighted by a recent analysis of whole-genome sequencing data that showed evidence of correlations between nuclear and mitochondrial alleles in specific geographic regions (van Oven and Kayser, 2009).

In this communication, we investigate mitonuclear associations using a GWAS approach with mitochondrial variants as the phenotype of interest. Using HapMap Phase 3 European sequence data, we selected variants with sufficient power to detect associations characterised by an odds ratio of at least 1.5 (Supplementary Materials). We selected two sets of European individuals genotyped on the same microarray system, one composed of patients with Parkinson’s disease (PD) and one composed of unselected individuals (58C/NBS, detailed in STable 1). For each cohort, we performed a genome-wide search for nuclear polymorphisms showing significant association with each of the selected mitochondrial variants. After examining single cohorts, we expanded our analyses to incorporate disease status by assessing the interaction between disease status and nuclear variants on the association for the combinations that achieved significance in at least one of the two cohorts. Finally, we quantified the effect of the combinations that showed a significant result in the last step on disease risk, i.e., the association between disease status and the interaction between mtSNP and nSNP.

## 2 Results

First, we determined the minor allele frequency (MAF) of the mtSNP that would allow us to achieve a power of 0.8 to detect an association with an odds ratio of 1.5, for a nominal type-I error of 5 × 10 ^−8^, a nSNP with a MAF of 0.10 and a cohort size of 2000 (Supplementary Materials). This required the mtSNP to have a MAF > 0.2. According to HapMap Phase 3 genome data, six mtSNPs met that threshold. However, three of these (mt.11467, mt.12308 and mt12372, STable 2) are strongly associated with each other (R^2^ > 0.9), with R^2^ < 0.5 for the remaining pairwise associations. Thus, we selected a subset of four mtSNPs (mt.2706, mt.3010, mt.11251 and mt.11467) for further analysis (Supplementary Materials).

Next, we assessed the associations between the nSNPs and the selected mtSNPs in each cohort using logistic regression (Supplementary Materials). For the unaffected individuals (58C/NBS; see Supplementary Materials), no mitonuclear association achieved a false discovery rate (FDR) below 0.05 (Q-Q plots for each mtSNP tested are shown in SFigure 1). Conversely, for PD, five combinations achieved this threshold (Table 1, Q-Q plots shown in SFigure 2 and locus plots in SFigures 3–5).

Next, we proceeded to investigate the modulation of these five mitonuclear associations by disease status using combined data from unaffected (58C/NBS) and affected (PD) individuals (Supplementary Materials). After logistic regression, the interaction term of disease and nSNP for all five combinations achieved p-values below 6.0 × 10 ^−5^, suggesting that the association between mtSNPs and nSNPs is influenced by disease status (Table 1).

Usually, interest focuses on the effect of mitonuclear combinations on disease risk, i.e., the effect of the interaction between mtSNP and nSNP on disease status (Kummer et al., 2019). Thus, we investigated the interaction terms for the five mitonuclear combinations (Supplementary Materials). They all achieved p-values < 7.0 × 10 ^−5^ and odds ratios ≥ 1.5, suggesting that the combination of mtSNP and nSNP alleles modify disease risk (Table 1).

## 3 Discussion

We began this study by using a single cohort approach to detect mitonuclear associations. We were able to identify such associations in a disease cohort but not amongst a control population, which may reflect the limited size of the cohort investigated. The results of previously published analyses are contradictory and do not always take population structure into account (Yamamoto et al., 2020; Yonova-Doing et al., 2021; van Oven and Kayser, 2009). Thus, a strength of the approach we adopted is that it allows us to account for population stratification using covariates.

The lack of significant associations among the unaffected cohort contrasts with the results we obtained for the PD cohort, where five mitonuclear combinations showed a significant association. These combinations were also associated with PD risk.

Two mtSNPs, mt.2706 and mt.11467, were involved in significant combinations and both define haplogroups (H and U respectively (Miyatake et al., 2018) that are associated with PD-risk (Hudson et al., 2013; Marom et al., 2017). However, in our data, these mtSNPs do not achieve a significant association with PD on their own (Table 1). The nuclear components of these combinations map to three different locations. The nuclear component of rs1606610-mt.11467, maps to the intergenic region, downstream of *AIPL1* (SFigure 5). However, the paucity of supporting SNPs in LD means this result should be interpreted with caution. For rs11666267-mt.2706, the nuclear variant maps to *NIBAN3* (SFigure 3). ClinVar includes a report associating this rare germline variant with cerebrovascular disease (ClinVar ID: SCV001338733). Further, the polymorphism is adjacent to the 5’UTR of *GLT25D1,* which encodes a collagen modifying enzyme, and an association with cerebrovascular disease has been reported (Kummer et al., 2019) and cerebrovascular disease has been postulated as a PD-risk factor (Tirozzi et al., 2021).

The nuclear components of the remaining significant combinations (all with mt.11467) map to *TBCA* (SFigure 4). *TBCA* encodes a tubulin chaperone and SNPs in the gene have been reportedly associated with PD age of onset (Stamper et al., 2008). In addition, its expression is altered in PD patients with dementia (Werner, 2008) and is elevated in the substantia nigra of PD patients (Spencer et al., 2011). In our study, the nuclear components of combinations mapping to *TBCA* achieved p-values between 10 ^−3^ and 6 ×10 ^−3^ (Table 1) in the association with PD, with odds ratios around 1.1.

It should also be noted that in a previous publication we identified several nSNPs showing associations with PD in the cohorts used here (Hamza et al., 2010; Gauderman, 2002) (SFigure 6). The p-values for the interactions of any of the mitonuclear combinations involving the lead SNPs (chr4 rs2736990, chr6 rs3094609 and chr17 rs393152) are all above 0.05.

Although our analyses are adequately powered to detect relatively large effects, our results should be replicated in a larger cohort for validation and to enable subtler associations to be detected. Nonetheless, we present an approach that allows us to identify associations between mitochondrial and nuclear variants whilst accounting for population stratification and show that mitonuclear combinations can contribute to Parkinson’s disease risk.

## Supplementary Material

Supplementary figures

Supplementary tables

## Figures and Tables

**Table 1:** Significant (FDR < 0.05) mitonuclear combinations identified in the PD cohort.

Mitonuclear combination						Mitonuclear association						Association with PD					
mtSNP			nSNP			in PD cohort			Effect of disease status on association^b^			mtSNP		nSNP		Mitonuclear combi nation^c^	
dbSNPID	chr:bp	Locus	ID	chr:bp	Locus	P	FDR	OR (95% CI)	Plot^a^	P	OR (95% CI)	P	OR (95% CI)	P	OR (95% CI)	P	OR (95% CI)
rs2854l28	mt.2706	*MT-RNR2*	rsl 1666267	19:17660300	*NIBAN3*	4.2E-08	l.5E-02	1.4 (1.3-1.6)	*SFig. 3*	2.5E-07	1.5 (0.1-1.7)	7.2E-02	0.6 (0.4-1.0)	2.1E-01	1.1 (1.0-1.1)	4.16E-06	1.5 (1.3-17)
rs2853493	mt.11467	*MT-ND4*	rs2544l2	5:77079452	*TBCA*	5.8E-09	1.0E-02	1.6 (1.4-1.8)	*SFig. 4*	1.1E-05	1.5 (1.3-1.8)	2.4E-01	0.9 (0.8-1.0)	1.1E-03	1.1 (1.0-1.2)	7.20E-05	1.5 (1.2-1.8)
rs2853493	mt.11467	*MT-ND4*	rs384109	5:77019909	*TBCA*	l.9E-08	l.2E-02	1.6 (1.3-1.8)	*SFig. 4*	l.2E-05	1.5 (1.3-1.8)	2.4E-01	0.9 (0.8-1.0)	2.1E-03	1.1 (1.1-1.2)	5.53E-05	1.5 (1.2-1.8)
rs2853493	mt. 11467	*MT-ND4*	rs44l492	5:76973352	*TBCA*	l.2E-08	1.1E-02	1.6 (1.3-1.8)	*SFig. 4*	5.7E-05	1.4 (1.2-1.7)	2.4E-01	0.9 (0.8-1.0)	6.9E-03	1.1 (1.1-1.2)	5.81E-05	1.5 (1.2-1.8)
rs2853493	mt. 11467	*MT-ND4*	rs1606610	17:6301562	*Interg*	3.6E-08	l.5E-02	1.7 (1.4-2.0)	*SFig. 5*	6.2E-06	1.7 (1.3-2.1)	2.4E-01	0.9 (0.8-1.0)	2.1E-01	1.1 (1.0-1.2)	9.19E-07	1.9 (1.5-2.4)

a Supplementary Figure reference;

b Assessed using the term for the interaction between disease status and nSNP;

c Assessed using the term for the interaction between mtSNP and nSNP; interg: Intergenic. Chromosome and position (chr:bp) are based upon GRCh37.

## References

[R1] Chinnery PF, Hudson G (2013). Mitochondrial genetics. Br Med Bull.

[R2] Gauderman WJ (2002). Sample size requirements for matched case-control studies of gene-environment interaction. Stat Med.

[R3] Ghezzi D, Marelli C, Achilli A, Goldwurm S, Pezzoli G, Barone P, Pellecchia MT, Stanzione P, Brusa L, Bentivoglio AR, Bonuccelli U (2005). Mitochondrial DNA haplogroup K is associated with a lower risk of Parkinson’s disease in Italians. Eur J Hum Genet.

[R4] Gonçalves VF, Giamberardino SN, Crowley JJ, Vawter MP, Saxena R, Bulik CM, Yilmaz Z, Hultman CM, Sklar P, Kennedy JL, Sullivan PF (2018). Examining the role of common and rare mitochondrial variants in schizophrenia. PLoS ONE.

[R5] Hamza TH, Zabetian CP, Tenesa A, Laederach A, Montimurro J, Yearout D, Kay DM, Doheny KF, Paschall J, Pugh E, Kusel VI (2010). Common genetic variation in the HLA region is associated with late-onset sporadic Parkinson’s disease. Nat Genet.

[R6] Hudson G, Keers S, Man PYW, Griffiths P, Huoponen K, Savontaus M-L, Nikoskelainen E, Zeviani M, Carrara F, Horvath R, Karcagi V (2005). Identification of an X-chromosomal locus and haplotype modulating the phenotype of a mitochondrial DNA disorder. Am J Hum Genet.

[R7] Hudson G, Nalls M, Evans JR, Breen DP, Winder-Rhodes S, Morrison KE, Morris HR, Williams-Gray CH, Barker RA, Singleton AB, Hardy J (2013). Two-stage association study and meta-analysis of mitochondrial DNA variants in Parkinson disease. Neurology.

[R8] Hudson G, Gomez-Duran A, Wilson IJ, Chinnery PF, Gojobori T (2014). Recent mitochondrial DNA mutations increase the risk of developing common late-onset human diseases. PLoS Genet.

[R9] Hyslop LA, Blakeley P, Craven L, Richardson J, Fogarty NME, Fragouli E, Lamb M, Wamaitha SE, Prathalingam N, Zhang Qi, O’Keefe H (2016). Towards clinical application of pronuclear transfer to prevent mitochondrial DNA disease. Nature.

[R10] Kang E, Wu J, Gutierrez NM, Koski A, Tippner-Hedges R, Agaronyan K, Platero-Luengo A, Martinez-Redondo P, Ma H, Lee Y, Hayama T (2016). Mitochondrial replacement in human oocytes carrying pathogenic mitochondrial DNA mutations. Nature.

[R11] Kivisild T (2015). Maternal ancestry and population history from whole mitochondrial genomes. Investig Genet.

[R12] Kummer BR, Diaz I, Wu X, Aaroe AE, Chen ML, Iadecola C, Kamel H, Navi BB (2019). Associations between cerebrovascular risk factors and parkinson disease. Ann Neurol.

[R13] Lopez Sanchez MIG, Kearns LS, Staffieri SE, Clarke L, McGuinness MB, Meteoukki W, Samuel S, Ruddle JB, Chen C, Fraser CL, Harrison J (2021). Establishing risk of vision loss in Leber hereditary optic neuropathy. Am J Hum Genet.

[R14] Marom S, Friger M, Mishmar D (2017). MtDNA meta-analysis reveals both phenotype specificity and allele heterogeneity: a model for differential association. Sci Rep.

[R15] Miyatake S, Schneeberger S, Koyama N, Yokochi K, Ohmura K, Shiina M, Mori H, Koshimizu E, Imagawa E, Uchiyama Y, Mitsuhashi S (2018). Biallelic COLGALT1 variants are associated with cerebral small vessel disease. Ann Neurol.

[R16] Pickett SJ (2018). Phenotypic heterogeneity in m.3243A>G mitochondrial disease: The role of nuclear factors. Ann Clin Transl Neurol.

[R17] Rollins B, Martin MV, Sequeira PA, Moon EA, Morgan LZ, Watson SJ, Schatzberg A, Akil H, Myers RM, Jones EG, Wallace DC (2009). Mitochondrial variants in schizophrenia, bipolar disorder, and major depressive disorder. PLoS ONE.

[R18] Samuels DC, Carothers AD, Horton R, Chinnery PF (2006). The power to detect disease associations with mitochondrial DNA haplogroups. Am J Hum Genet.

[R19] Sloan DB, Fields PD, Havird JC (2015). Mitonuclear linkage disequilibrium in human populations. Proc Biol Sci.

[R20] Spencer CCA, Plagnol V, Strange A, Gardner M, Paisan-Ruiz C, Band G, Barker RA, Bellenguez C, Bhatia K, Blackburn H, Blackwell JM (2011). Dissection of the genetics of Parkinson’s disease identifies an additional association 5’ of SNCA and multiple associated haplotypes at 17q21. Hum. Mol Genet.

[R21] Stamper C, Siegel A, Liang WS, Pearson JV, Stephan DA, Shill H, Connor D, Caviness JN, Sabbagh M, Beach TG, Adler CH (2008). Neuronal gene expression correlates of Parkinson’s disease with dementia. Mov Disord.

[R22] Tirozzi A, Parisi R, Cerletti C, Donati MB, de Gaetano G, Iacoviello L, Gialluisi A (2021). Genomic Overlap between Platelet Parameters Variability and Age at Onset of Parkinson Disease. Applied Sciences.

[R23] Tranah GJ, Santaniello A, Caillier SJ, D’Alfonso S, Martinelli Boneschi F, Hauser SL, Oksenberg JR (2015). Mitochondrial DNA sequence variation in multiple sclerosis. Neurology.

[R24] van Oven M, Kayser M (2009). Updated comprehensive phylogenetic tree of global human mitochondrial DNA variation. Hum Mutat.

[R25] Vyshkina T, Banisor I, Shugart YY, Leist TP, Kalman B (2005). Genetic variants of Complex I in multiple sclerosis. J Neurol Sci.

[R26] Wei W (2019). Germline selection shapes human mitochondrial DNA diversity. Science.

[R27] Werner CJ (2008). Proteome analysis of human substantia nigra in Parkinson’s disease. Proteome Sci.

[R28] Yamada M, Emmanuele V, Sanchez-Quintero M, Sun B, Lallos G, Paull D, Zimmer M, Pagett S, Prosser R, Sauer M, Hirano M (2016). Genetic Drift Can Compromise Mitochondrial Replacement by Nuclear Transfer in Human Oocytes. Cell Stem Cell.

[R29] Yamamoto K, Sakaue S, Matsuda K, Murakami Y, Kamatani Y, Ozono K, Momozawa Y, Okada Y (2020). Genetic and phenotypic landscape of the mitochondrial genome in the Japanese population. Commun Biol.

[R30] Yonova-Doing E, Calabrese C, Gomez-Duran A, Schon K, Wei W, Karthikeyan S, Chinnery PF, Howson JMM (2021). An atlas of mitochondrial DNA genotypephenotype associations in the UK Biobank. Nat Genet.

