# Supplementary material for: Interactions between nuclear and mitochondrial SNPs and Parkinson’s disease risk
Source: Mitochondrion. Author manuscript; Available in PMC 2024 Dec 9. (PMC7617118; doi:10.1016/j.mito.2022.02.002)
Supplement: Supplementary figures [file EMS181811-supplement-Supplementary_figures.pdf]

Index

SFigure 1 | QQ plots of mitonuclear associations in 58C/NBS combined.

SFigure 2 | QQ plots of mitonuclear associations in PD

SFigure 3 | LocusZoom plot of the region containing rs11666267.

SFigure 4 | LocusZoom plot of the region containing rs254412, rs384109 and rs441492.

SFigure 5 | LocusZoom plot of the region containing rs1606610.

SFigure 6 | SFigure 10 | QQ plot of PD versus controls.

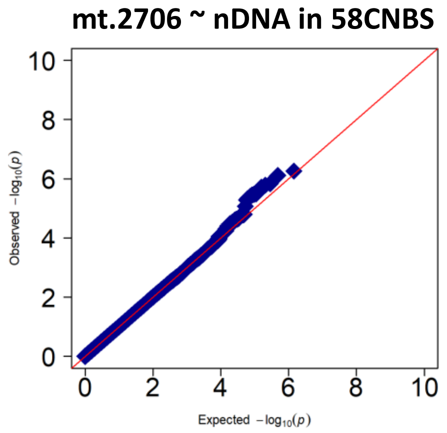

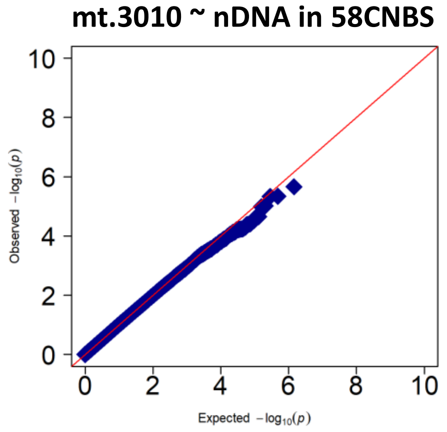

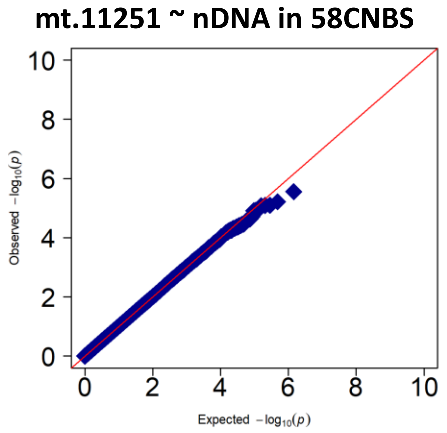

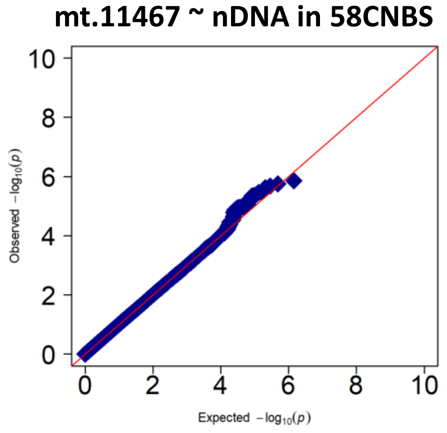

SFigure 1 | QQ plots of mitonuclear associations in 58C/NBS cohorts combined.
QQplots are shown for each of the selected mtSNPs (mt.2706, mt.3010, mt.11251 and mt.11467) in combined as 58C/NBS. Note, no nSNP reached achieved a P-value of <5x10-8.

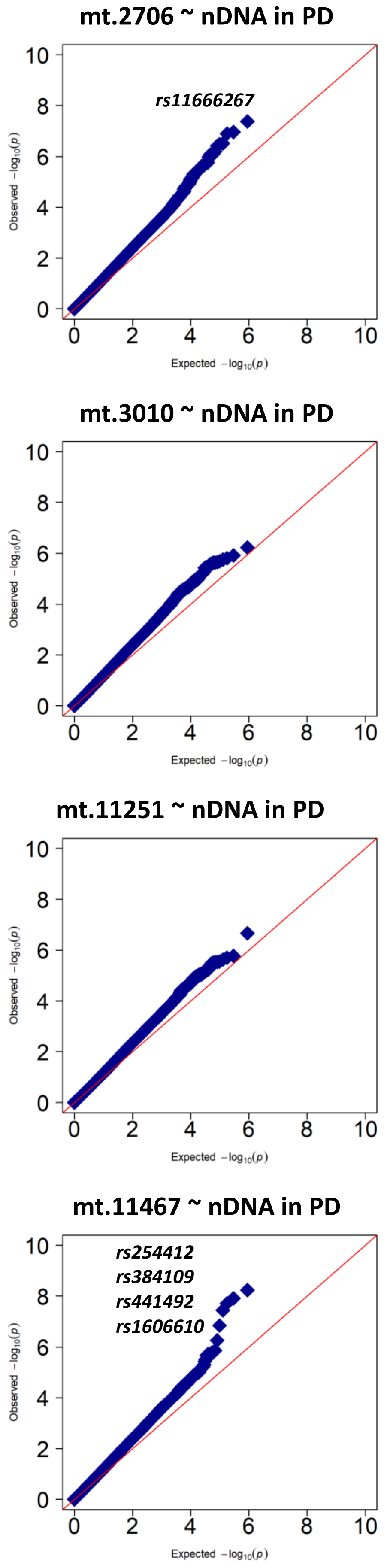

SFigure 2 | QQ plots of mitonuclear associations in PD

QQplots are shown for each of the selected mtSNPs (mt.2706, mt.3010, mt.11251 and mt.11467) in PD. Note, nSNPs achieving a P-value of <5x10-8 are highlighted.

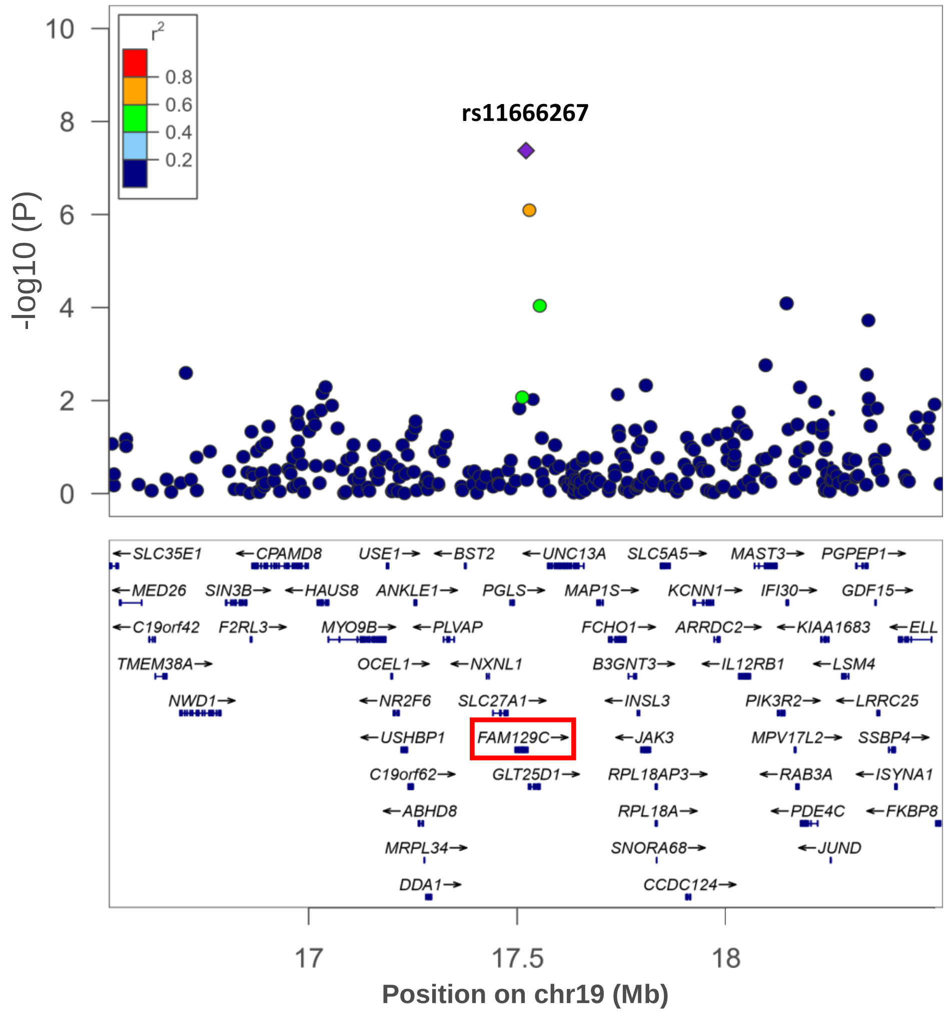

SFigure 3 | LocusZoom plot of the region containing rs11666267.

LocusZoom plot of the region (+/- 1000KB) surrounding rs11666267 (**Table 1**). R^2 determined using HapMap CEU samples. FAM129C is also known as NIBAN3.

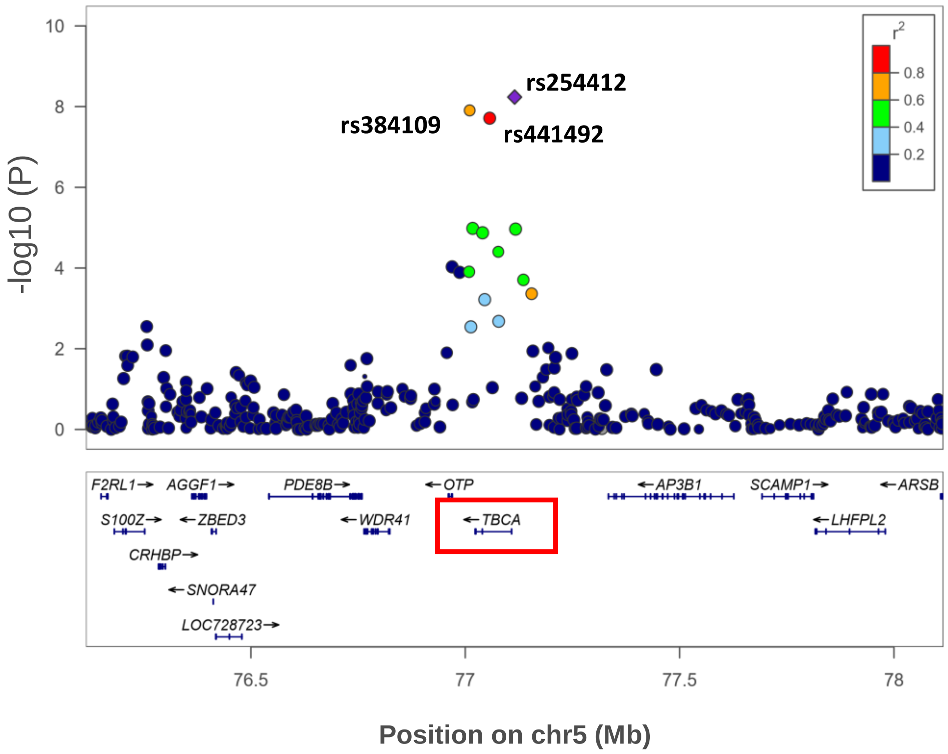

SFigure 4 | LocusZoom plot of the region containing rs254412, rs384109 and rs441492.

LocusZoom plot of the region (+/- 1000KB) surrounding rs254412, rs384109 and rs441492 (**Table 1**). R^2 determined using HapMap CEU samples.

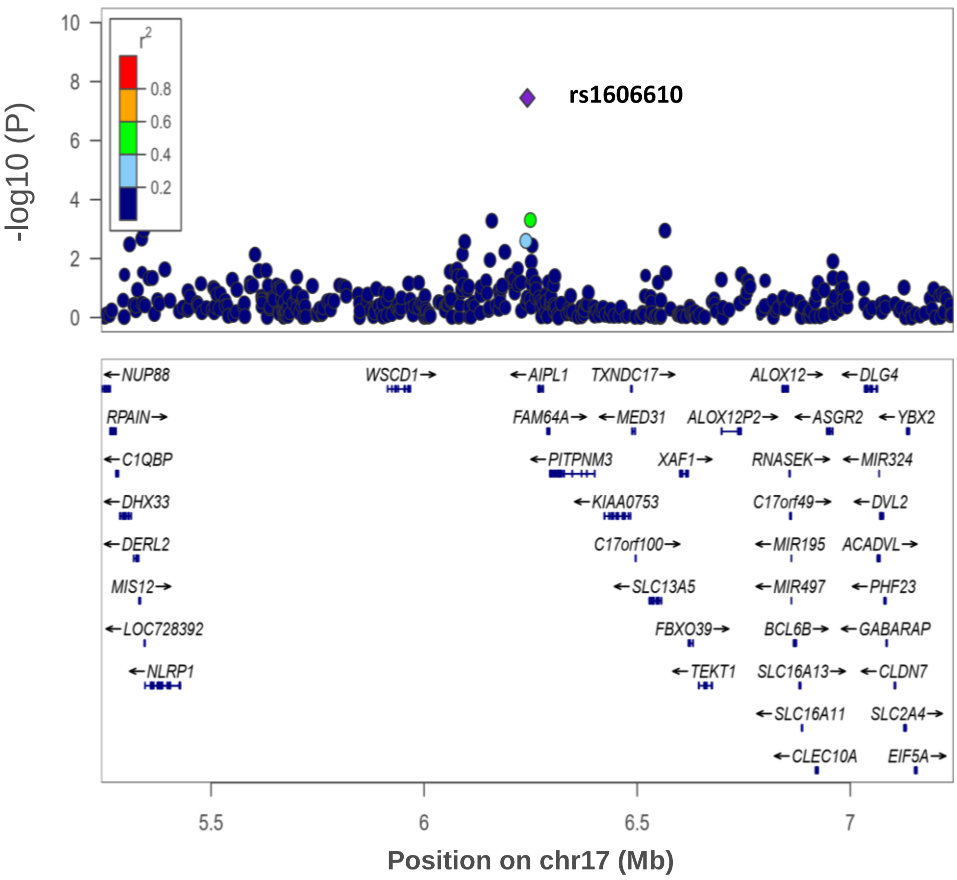

SFigure 5 | LocusZoom plot of the region containing rs1606610.

LocusZoom plot of the region (+/- 1000KB) surrounding rs1606610 (Table 1). R^2 determined using HapMap CEU samples.

a)

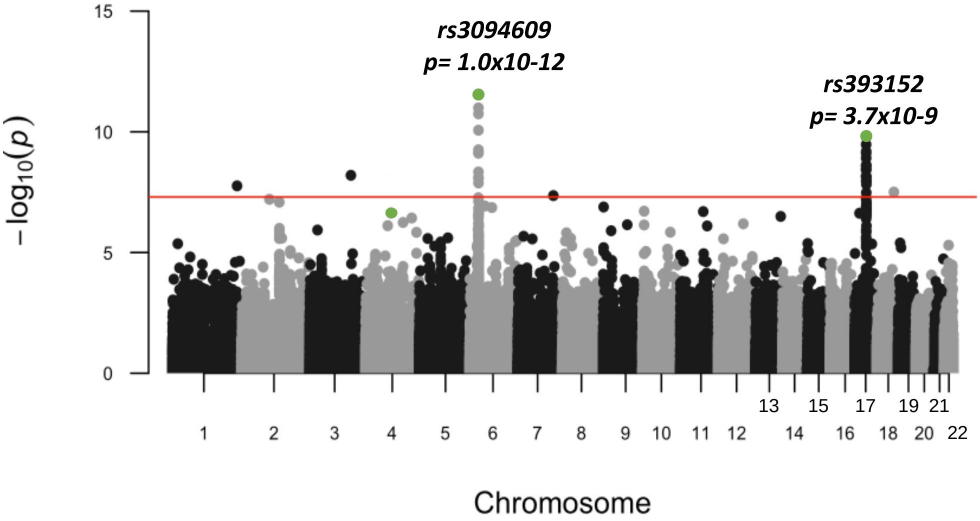

a)

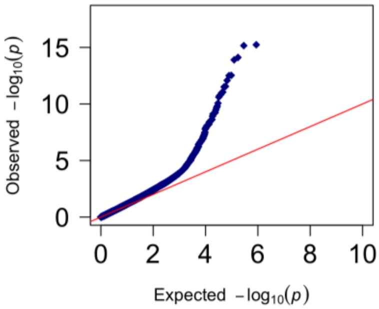

SFigure 6 | nSNPs in PD versus Controls (58CNBS).

Manhattan plot (a) and corresponding Q-Q plot (b) of the association between nSNPs in PD cases versus controls (58CNBS). The Q-Q plot shows significant ($5.0x10^{-8}$) association between PD and 55 nSNPs, which is inline with previous observations.
